# Biological Effects of “Inflammageing” on Human Oral Cells: Insights into a Potential Confounder of Age-Related Diseases

**DOI:** 10.3390/ijms25010005

**Published:** 2023-12-19

**Authors:** Elli Alexakou, Athina Bakopoulou, Danae A. Apatzidou, Aristeidis Kritis, Andigoni Malousi, Vassiliki Anastassiadou

**Affiliations:** 1 Department of Prosthodontics, School of Dentistry, Faculty of Health Sciences, Aristotle University of Thessaloniki (A.U.TH.), 54124 Thessaloniki, Greece; ellialexa@yahoo.gr (E.A.); vanasta@dent.auth.gr (V.A.); 2 Department of Preventive Dentistry, Periodontology & Implant Biology, School of Dentistry, Faculty of Health Sciences, Aristotle University of Thessaloniki (A.U.TH.), 54124 Thessaloniki, Greece; dapatzidou@dent.auth.gr; 3 Department of Physiology, School of Medicine, Faculty of Health Sciences, Aristotle University of Thessaloniki (A.U.TH.), 54124 Thessaloniki, Greece; kritis@auth.gr; 4 Regenerative Medicine Center, Basic and Translational Research Unit (BTRU) of Special Unit for Biomedical Research and Education (BRESU), Faculty of Health Sciences, School of Medicine, Aristotle University of Thessaloniki, 54124 Thessaloniki, Greece; 5 Department of Biological Chemistry, School of Medicine, Faculty of Health Sciences, Aristotle University of Thessaloniki (A.U.TH.), 54124 Thessaloniki, Greece; andigoni@auth.gr

**Keywords:** inflammageing, senescence, pro-inflammatory stimulus, senescence-associated secretory phenotype

## Abstract

Objectives: The term “inflammageing” describes the process of inflammation-induced aging that leads living cells to a state of permanent cell cycle arrest due to chronic antigenic irritation. This in vitro study aimed to shed light on the mechanisms of “inflammageing” on human oral cells. Methods: Primary cultures of human gingival fibroblasts (hGFs) were exposed to variable pro-inflammatory stimuli, including lipopolysaccharide (LPS), Tumor Necrosis Factor-alpha (TNFa), and gingival crevicular fluid (GCF) collected from active periodontal pockets of systemically healthy patients. Inflammageing was studied through two experimental models, employing either late-passage (“aged”) cells (p. 10) that were exposed to the pro-inflammatory stimuli or early-passage (“young”) cells (p. 1) continuously exposed during a period of several passages (up to p. 10) to the above-mentioned stimuli. Cells were evaluated for the expression of beta-galactosidase activity (histochemical staining), senescence-associated genes (qPCR analysis), and biomarkers related to a Senescence-Associated Secretory Phenotype (SASP), through proteome profile analysis and bioinformatics. Results: A significant increase (*p* < 0.05) in beta-galactosidase-positive cells was observed after exposure to each pro-inflammatory stimulus. The senescence-associated gene expression included upregulation for *CCND1* and downregulation for *SUSD6*, and *STAG1*, a profile typical for cellular senescence. Overall, pro-inflammatory priming of late-passage cells caused more pronounced effects in terms of senescence than long-term exposure of early-passage cells to these stimuli. Proteomic analysis showed induction of SASP, evidenced by upregulation of several pro-inflammatory proteins (IL-6, IL-10, IL-16, IP-10, MCP-1, MCP-2, M-CSF, MIP-1a, MIP-1b, TNFb, sTNF-RI, sTNF-RII, TIMP-2) implicated in cellular aging and immune responses. The least potent impact on the induction of SASP was provoked by LPS and the most pronounced by GCF. Conclusion: This study demonstrates that long-term exposure of hGFs to various pro-inflammatory signals induced or accelerated cellular senescence with the most pronounced impact noted for the late-passage cells. The outcome of these analyses provides insights into oral chronic inflammation as a potential confounder of age-related diseases.

## 1. Introduction

The term “Inflammageing” describes the process of inflammation-induced aging that leads living cells to a state of permanent cell cycle arrest due to chronic antigenic irritation. It was first reported by Franceschi et al. in 2000 and was very early associated with age-related diseases. Inflammageing has attracted the interest of the scientific community early on, resulting in the development of various concepts that try to explain and approach the biological process that takes place [1]. In essence, inflammageing is initiated in a microenvironment with elevated levels of pro-inflammatory and reduced levels of anti-inflammatory cytokines, which leads to a low-grade, asymptomatic chronic and systemic inflammatory response of the host. The above overactive reaction is maintained for an extended period after the removal of the initial stimulus [2,3,4]. During this process, a series of complex responsive events take place. It is essentially an interaction between the cells and elements of the microenvironment, aiming to adjust the balance between physiological and pathological signaling networks. Inflammageing represents a non-resolving state of the inflammatory response of the host, where the presence of an insistent and low-intensity stimulation and long-term responses in target tissues leads to a failure of the immune system to restore the equilibrium between pro-resolution and pro-inflammatory mediators [1,5]. This has as a direct consequence the decline of many mechanisms related to the ability of the tissues for healing and defense, therefore perturbing any therapeutic protocols for tissue engineering and regeneration.

In the field of Geroscience, inflammation has been considered as one of the seven evolutionarily conserved mechanistic pillars of ageing that are common in age-related diseases. In the oral cavity, it has been confirmed that persistent local inflammation affects and even triggers the onset of systemic diseases, such as diabetes, cardiovascular disease, and dementia, and has a negative impact on nutrition, well-being, and overall quality of life (QoL) [6,7,8]. Oral pathogenic bacteria associated with periodontitis, caries, and other metabolites of the oral microbiome can enter the systemic circulation through the periodontal blood and bloodstream and consequently affect the systemic health of the human body. [9,10,11]. Besides, inflammageing is the long-term result of the chronic physiological stimulation of the innate immune system, which can become damaging during ageing, a period of life largely unpredicted by evolution [12].

Ageing can be characterized as a progressive physiological change in an organism leading to a decline of several biological functions, cellular senescence, and finally loss of the ability of the organism for adaptation and homeostasis [13,14]. New findings suggest that ageing is a modifiable risk factor, and, in this respect, it may be feasible to delay age-related diseases by modulating fundamental aging mechanisms. One of these mechanisms is cellular senescence, which refers to the biological ageing of the living cells, that may further initiate inflammageing. The increasing load of senescent cells results in the release of soluble agents, like cytokines, chemokines, growth factors, and enzymes proteases, into the surrounding microenvironment, a phenomenon known as “Senescence-Associated Secretory Phenotype” (SASP), which eventually contributes to tissue dysfunction. SASP-mediated microenvironmental stimuli may induce a “pro-inflammatory licensing” to the neighboring healthy cells, initiating a “vicious cycle” and leading to a further increase in inflammation and dysfunction. The proteins associated with the SASP, such as TNFa, IL-6, monocyte chemoattractant proteins (MMPs), proteins-1 (MCP-1), and IGF-binding proteins (IGFBPs), grow in multiple chronologically aged tissues and induce initiation and evolution of aseptic inflammation [15,16]. Conversely, a local, time-limited SASP may be important for resolving tissue damage, at least in younger adults, as it can alert adjacent cells to potential risk and promote the immune clearance of damaged cells [17,18].

Inflammageing has been barely studied in conjunction with conditions prevailing in the oral cavity and its impact on oral cells and tissues. Based on the above, the present study aimed to explore the effects of various pro-inflammatory signals on the induction and/or acceleration of cellular senescence and the acquirement of an SASP phenotype by human oral cells. This study was based on two pillars and a panel of methods was used to collect the data. The assessment of cellular senescence relied on determining the presence of beta-galactosidase-positive cells, a widely utilized experimental method for evaluating aging. Additionally, real-time PCR analysis of senescence-associated genes (*CCND1*, *SUSD6*, *STAG1*) was conducted. The abundance of SASP-related markers was further evaluated based on the proteomics analysis. The goal was to identify the cellular senescence-related biomarkers that drive inflammageing in the oral cavity and can therefore be used as indicators of an increase in vulnerability toward systemic health effects. The null hypotheses of this study were that exposure of healthy human gingival oral cells to pro-inflammatory stimuli does not affect the progress of cellular senescence and does not promote the acquirement of SASP.

## 2. Results

### 2.1. Senescence-Related Beta-Galactosidase Activity

**Figure 1:**
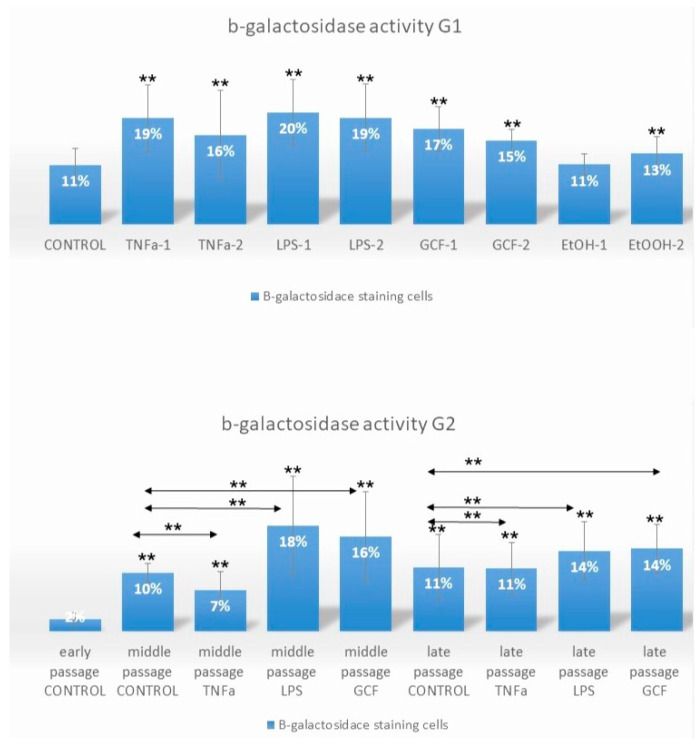
Impact of long-term and short-term exposure of HGFs to several pro-inflammatory signals (TNFa, LPS, GCF) on β-galactosidase activity. Percentage of SA-β-gal-positive HGFs cells exposed short-term at late-passage G1 (TNFa-1 = 0.1 μL/mL, TNFa-2 = 1 μL/mL, LPS-1 = 0.2 μL/mL, LPS-2 = 1 μL/mL, GCF-1 = 5 μL/mL, GCF-2 = 10 μL/mL, EtOH-1 = 100 mM, EtOH-2 = 500 mM) and long-term, from early to late passages, G2 (TNFa = 1 μL/mL, LPS = 0.2 μL/mL, GCF = 10 μL/mL) to pro-inflammatory signals. Values are mean (±SD) of HGFs (experiments repeated three times in duplicates). Asterisks indicate statistically significant differences (** *p*  <  0.01) between control group (G1) vs. all the signals groups and early control group (G2) vs. middle and late passages for each signal separately. The horizontal double arrows indicate statistically significant differences between HGFs expanded in the same passage group. Statistical analyses were performed by two-way ANOVA followed by Tukey’s post hoc tests.

The Senescence-related beta-galactosidase activity (SA-beta-gal) in Human Gingival Fibroblast (HGF) cultures was evaluated either in late-passage cells after short-term exposure to pro-inflammatory signals (G1) at different concentrations or after prolonged exposure of early-passage cells from p. 2 to p. 10 to the same signals (G2) at one concentration per signal (Figure 1, Supplementary Figure S1, see Materials and Methods for details of treatments in each group—G). In the G1 case, a significant increase in SA-beta-gal-positive cells (** *p* < 0.01) was observed after exposure to all pro-inflammatory signals, except the lowest concentration ethanol (EtOH-1 group = 100 mM). In the G2 case, the highest number of SA-beta-gal-positive cells was observed at middle passages for the LPS- (** *p* < 0.01) and GCF (** *p* < 0.01), the least number for the TNFa-exposed cells (** *p* < 0.01), and the same trend was preserved also for late-passage cells (** *p* < 0.01).

### 2.2. Senescence-Related Gene Expression Patterns

**Figure 2:**
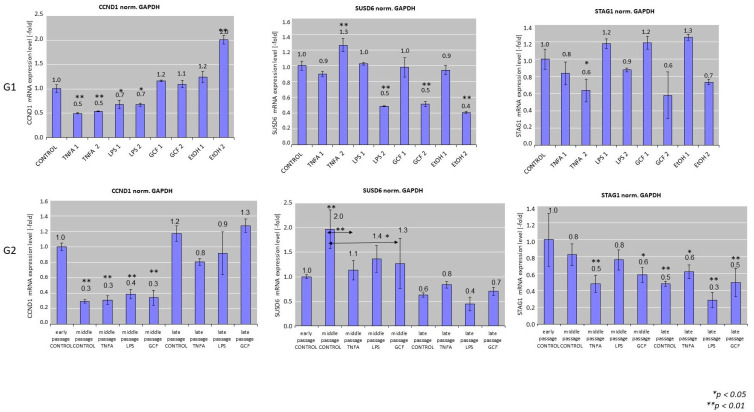
Real-time polymerase chain reaction (PCR) analysis of the expression of senescence-related genes, including *CCND1*, *SUSD6*, *STAG1* in HGFs cultures exposed to several pro-inflammatory signals, including LPS, TNFa, and GCF. Human Gingival Fibroblasts (HGFs) without treatment were used as controls and GAPDH as the housekeeping gene for this assay. EtOH was used at G1 setup as positive control group. Real-time PCR values are means ± standard deviations of three independent setups in duplicates (* *p* < 0.05, ** *p* < 0.01 compared with control untreated cultures). The double arrows (G2) indicate statistically significant differences between groups expanded in the same passage.

Figure 2 depicts the results of senescence-related gene expression for cells exposed to various pro-inflammatory signals. In specific, gene expression analysis by real-time PCR after short-term exposure of late-passage cells to pro-inflammatory signals (G1) showed statistically significant downregulation of *CCND1* in the TNFa group (0.5 ± 0.1-fold) compared to the control for both concentrations (TNFa-1 and TNFa-2, ** *p* < 0.01), and in the LPS group (0.7 ± 0.1-fold) compared to the control for both concentrations (LPS-1 and LPS-2, ** *p* < 0.01) and statistically significant upregulation in the EtOH-2 group (2.0 ± 0.1-fold, ** *p* < 0.01). In all other groups, no statistical difference was observed.

*SUSD6* and *STAG1* showed a trend for downregulation after exposure to increasing concentrations of the pro-inflammatory factors. Regarding *SUSD6*, a statistically significant decrease was observed in the LPS-2 (0.5 ± 0.0-fold, ** *p* < 0.01), GCF-2 (0.5 ± 0.0-fold, ** *p* < 0.01), and EtOH-2 (0.4 ± 0.0-fold, ** *p* < 0.01) groups, while a statistically significant increase was observed in the TNFa-2 group (1.3 ± 0.2-fold, ** *p* < 0.01). Expression of *STAG1* showed a downregulation in the TNFa-2 (0.6 ± 0.1-fold, * *p* < 0.05) and GCF-2 (0.6 ± 0.3-fold, * *p* < 0.05) groups.

Senescence-related gene expression patterns after long-term exposure of early-passage cells to pro-inflammatory signals (G2) showed a statistically significant (*p* < 0.01) downregulation of *CCND1* expression in all groups including the control in middle passages (approx. 0.3 ± 0.1-fold for all groups) compared to the early-passage control group, followed by an increase in gene expression at late-passage groups, but without any differences within the same passage group.

*SUSD6* expression levels showed a general reduction between the different stimuli groups at every passage. A statistically significant downregulation of *SUSD6* expression was observed in the middle-passage control group compared to the early-passage control group (2.0 ± 0.4-fold, ** *p* < 0.01), while significant downregulation compared to the respective middle-passage control was observed for the TNFa group (1.1 ± 0.2-fold, ** *p* < 0.01) and the GCF group (1.3 ± 0.5-fold, ** *p* < 0.01). Cells at late passage showed an overall downregulation of *SUSD6* expression, but without any differences within the same late passage between the experimental groups.

*STAG1* gene expression showed a decline in the G2 scenario. Statistically significant downregulation appeared in the TNFa group (0.5 ± 0.1-fold, ** *p* < 0.01) and GCF group (0.6 ± 0.1-fold * *p* < 0.05) at middle passages and in all groups at late passages compared to the early-passage control group. No differences were observed between groups within the same passage.

### 2.3. SASP-Related Marker Expression Analysis

**Figure 3:**
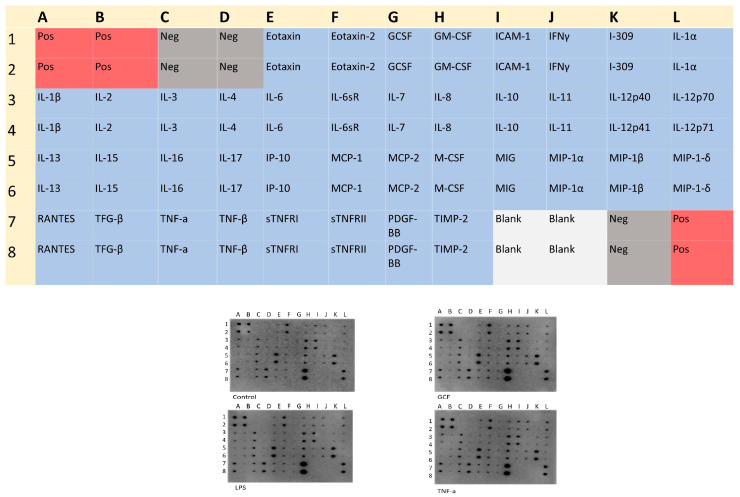
Human Inflammation Antibody Array Membrane Map of 40 inflammatory factors and the corresponding dot blots in each exposure pro-inflammatory signal (GCF, LPS, TNFa) compared to the control group. Letters and numbers on the dot plot correspond to the inflammatory factors shown in the map above.

**Figure 4:**
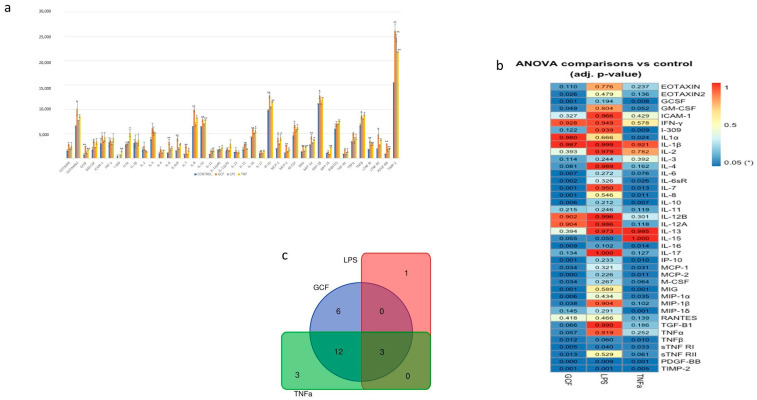
(**a**) Semi-quantitative analysis of SASP-marker expression by Image J. After normalization to positive control signal intensities, the comparison of the relative expression levels, analyte-by-analyte, among the groups followed. By comparing these signal intensities, the relative differences in cytokine expression were determined in each sample. (**b**) Heat-map of the ANOVA-adjusted *p*-values for pairwise comparisons between GCF, LPS, and TNFa vs. control. (**c**) Number of statistically significant proteins (adj. *p*-value ≤ 0.05) overlapping in GCF, LPS, and TNFa experiments (* *p* < 0.05, ** *p* < 0.01).

The semi-quantitative analysis, which was performed by proteome antibody-based arrays (Figure 3), showed that the abundance of several inflammatory molecules presented statistically significant differences among the experimental groups. Semi-quantitative analysis of the dot-blots among the experimental groups revealed statistically significant differences for 21 proteins in the GCF group, 4 proteins in the LPS group, and 18 proteins at the TNFa group compared all to control groups (Figure 4). Among the significantly modulated cytokines, several pro-inflammatory proteins (e.g., IL-6, IL-10, IL-16, IP-10, MCP-1, MCP-2, M-CSF, MIP-1a, MIP-1b, TNFb, sTNF-RI, sTNF-RII, TIMP-2) implicated in cellular aging and immune responses were significantly upregulated in all groups. Overall, the smallest impact on SASP was incited by LPS and the most explicit by GCF.

**Figure 5:**
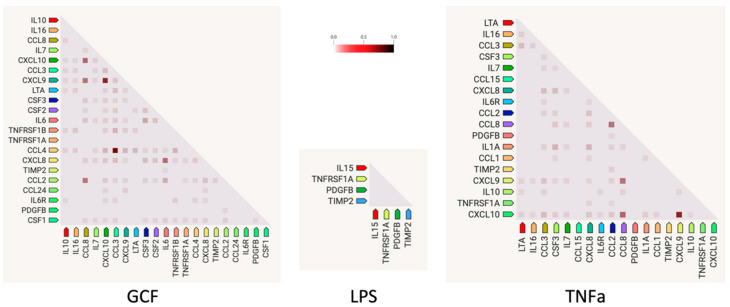
Gene co-expression analysis shows the level of confidence that two proteins are functionally associated, given the overall gene expression data in humans. The color intensity in the squares represents the level of association of the expression data, ranging between 0 (no association confidence) and 1 (high association confidence).

Gene co-expression analysis (Figure 5) shows the level of confidence that two proteins are functionally associated, given the overall gene expression data in humans. Based on the RNA-expressing levels in humans, the higher co-expression score was observed for the pairs CCL3-CCL4 (score: 0.974) for the GCF- and CXCL9–CXCL10 (score: 0.848) for both GCF- and TNFa groups.

In addition, the protein–protein interaction network analysis was constructed using the STRING-DB database and the connection/interaction between the proteins that were found statistically significant in each stimulus were depicted in diagrams (Supplementary Figure S2).

**Figure 6:**
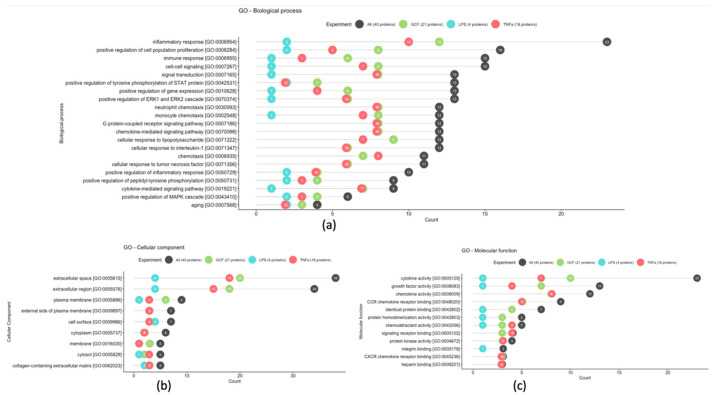
Most frequent gene ontology terms among statistically significant GCF, LPS, and TNFa proteins based on the biological process (**a**), cellular component (**b**), and molecular function (**c**) GO terms.

Finally, Gene Ontology (GO) enrichment analysis was used to identify the biological roles and functions of specific genes and their products. Differentially expressed proteins were annotated based on the terms of the biological process, molecular function, and cellular component GO categories (Figure 6). In terms of biological processes, most of the protein class, 23 in number, is associated with inflammatory response and 4 of them are associated with the aging process. For the cellular component, most proteins were distributed in the extracellular space and region (38 and 34 in number, respectively). The proteins of molecular function, 23 in number, were mainly involved in cytokine activity.

## 3. Discussion

The current study shed light into how the induction of mild, pro-inflammatory signals may affect the acceleration of senescence and the development of SASP in human oral cells. Based on the results of the experimental processes, both null hypotheses are rejected.

Various pro-inflammatory signals (TNFa, LPS, and GCF) known to predominate in inflammatory conditions in the oral cavity, including caries and periodontal disease, have been experimentally evaluated in terms of their potential to induce cellular senescence and to provoke a microenvironment suitable for the induction of SASP. In particular, tumor necrosis factor-alpha (TNFa) is a pleiotropic cytokine, characterized as a main regulator of inflammatory responses, while its presence in high amounts is associated with the pathogenesis of several inflammatory diseases, like Crohn’s disease and rheumatoid arthritis [19,20]. Lipopolysaccharide (LPS) is a representative component of the membrane of gram-negative oral bacteria and has been studied for its ability to induce a pro-inflammatory phenotype in various somatic cell types, such as osteocytes and cells derived from human gingival fibroblasts [21,22]. Gingival crevice fluid (GCF) is an inflammatory exudate that has been collected from deep periodontal pockets of systemically healthy adults suffering from periodontitis [23]. This fluid mainly consists of serum, tissue-collapsed products, inflammatory mediators, and antibodies that fight oral bacterial infections [24]. EtOH, which has been used in the present study as a positive control for induction of cellular senescence, is an ingredient of many products that come into contact with the oral mucosa, such as drugs and mouthwashes. This has led many researchers dealing with its effect on oral cells to conclude that while small concentrations may be beneficial for healing, large concentrations for a long period of exposure may be toxic, affecting cell proliferation, morphology, and viability [25,26,27]. The exposure concentrations of the pro-inflammatory signals mentioned above were selected in the present study based on their use in previous experiments reported in the literature, as being effective in inducing a pro-inflammatory environment without causing cell toxicity [22,27,28,29].

The evaluation of cellular senescence was based on the quantification of beta-galactosidase-positive cells, a commonly used experimental procedure for assessing aging, as well as on real-time PCR analysis of senescence-related genes. Even though increased numbers of SA-β-gal-positive cells were observed by the progression of passages, the number was statistically higher for the cells exposed to the pro-inflammatory signals. The increased number of SA-gal-positive cells with passaging was to be expected, as the gradual decline of cell division in growing cultures is a known process for all human cell populations unless the cells are immortalized [30,31]. Nevertheless, subsequent discoveries have shown that senescence can also be provoked as a cellular reaction to DNA damage. This response is triggered by external influences on cells, like oxidizing agents, oncogenic signals, tumorigenic factors, such as transforming growth factor-β (TGF-β) and TNFa, or bacterial infections and accumulations, like LPS induction [32].

Regarding the effect of TNFa and LPS on cellular senescence, this has been confirmed by previous studies in various cell lines, like mesenchymal stem cells, osteocytes, and human gingival fibroblasts [21,33,34,35]. The results of the present study showed that in both long-term and short-term exposure there is an increase in the proportion of positive cells for SA β-gal. Also, the number of senescent cells at the long-term exposure conditions was found to show a downward trend in the latter passages, since various mechanisms that affect the proliferation and cycling of the cells take place. Research has shown that the application of substances, like LPS or TNFa, leads to a decrease in telomere length [36]. It also results in an increase in the expression of molecules associated with cell cycle arrest (p16/INK4, p21/WAF-1, and p53) and inflammatory molecules (ICAM-1, IL-1β, IL-6, IL-8, MCP-1, MMP12, MMP13) [21]. Additionally, genes linked to inflammation (TNFa, IL-6, MCP-1) are upregulated, and signaling molecules related to SASP, such as C/EBPβ, p38 MAPK, and NF-κB p65, become activated. This results in increased expression of molecules associated with cell cycle arrest (p16/INK4, p21/WAF-1, and p53) and inflammatory responses (production of IL-6, TNF-α, and CXCL1) [37]. Furthermore, there is an elevation in the production of reactive oxygen species (ROS) [38,39]. Depending on the specific cell type and culture conditions, these effects lead to reduced proliferation rates and a shift in the culture composition toward senescent cells [40,41]. What is more, SA-β-gal activity, although indicated, is neither causal nor specific for aging and is mainly associated with a non-proliferative state of the cells [42,43]. For this reason, it should be interpreted in conjunction with other biomarkers, for example, secretion of bio-active factors related to SASP or real-time PCR analysis of the expression of senescence-related genes [44].

Among several senescence-specific genes reported in the literature, *Cyclin D1* (*CCND1*), *SUSD6*, and *STAG1* were selected. *Cyclin D1* is a regulatory subunit of CDK4 or CDK6, whose activity is required for the G1/S transition. *SUSD6* is involved in cell death and cellular response to DNA damage stimulus; while *STAG1* is a component of the cohesin complex, which is required for the cohesion of sister chromatids after DNA replication [45]. The above-mentioned genes are directly or indirectly associated with cellular functions implicated in the progression of senescence, such as cell proliferation, DNA replication, growth, differentiation, glucose transportation, or cell death. In addition, these genes are specific for senescence and not for other conditions, such as cell quiescence or cell cycle arrest. Gene expression in senescent cells is dynamic and the transcriptome of senescent cells is highly heterogeneous and associated with the senescence induction conditions (e.g., replicative senescence, oncogene-induced senescence, ionizing radiation-induced senescence, oxidative stress, etc.), as well as the cell type (e.g., fibroblasts, keratinocytes, melanocytes) [46,47]. Many studies that focused on cellular senescence report gene regulation 7 to 10 days after the application of various senescence inducers (e.g., replicative senescence, TNFa-induced senescence). However, this period may not be sufficient or may not be indicative for all cell types, such as in fibroblasts, which have shown a statistically significant alteration in senescence-related gene expression after 10 days of exposure [39,48].

Based on the above, the present study performed not only short-term exposure of late-passage gingival fibroblasts to pro-inflammatory signals, but also a long-term exposure of these cells from early up to late passage to these stimuli. The latter is recapitulating the continuous exposure of the oral cavity to such stimuli even in young patients suffering chronic inflammatory oral diseases, such as caries or periodontal disease (e.g., gingivitis or juvenile periodontitis). The importance of extending the period of study in the expression of senescence-specific genes in oral gingival cells lies in the fact that they show various significant ups and downs in expression throughout the evaluation period. For example, while they may initially show an upregulation in expression until the middle passages, this may change reaching the late passages where a downregulation of expression is observed, or vice versa [46,47,49,50]. Noteworthy, the increase in the cycling D1 has been directly related with senescence. An increase in Cyclin expression, in combination with the increase in the period of the signals exposure, is indicative of a gradual decrease in cell proliferation and cycling [51,52]. In addition, research that has been done on the expression of the *SUSD6* gene reports that its overexpression represses the progression of the cell cycle at the G2/M phase and causes a decrease in the expression of cyclin [53,54]. This opposite trend in expression was also evident in the current experiment in the long-term exposure, where in the middle passages an increase in *CCND1* was observed with a decrease in *SUSD6*, while in the later passages, the opposite trend was observed. Thus, it becomes apparent that a more senescence prevailing profile of the cultures dominates over time, since the most robust cells manage to survive.

An additional method used in the present study to correlate the existence of inflammation with aging was the proteomic analysis. This was carried out using antibody microarray, a technique that is widely used in biomedical research due to several advantages over other commonly used techniques, such as enzyme-linked immunosorbent assays (ELISA) and Western blotting. The use of antibody microarrays is a sensitive technique, which requires small sample volumes, and easy and friendly experimental procedures, and it takes less than 24 h to complete the process and capture the results [55].

Elevated levels of pro-inflammatory mediators, such as IL-6, IL-10, IL-16, IP-10, MCP-1, MCP-2, M-CSF, MIP-1a, MIP-1b, TNFb, sTNF-RI, sTNF-RII, TIMP-2 were observed in all experimental groups with the most pronounced differences in cells exposed for long-term to GCF. The latter could be expected, as this pro-inflammatory cocktail constitutes a complex mixture of numerous inflammatory elements, cytokines, metabolic and bacterial products, as already reported in many studies in the past and also verified by the analysis of the GCF used in the present study, which contained significant amounts of IL-10, BMP-7, TNFa, RANKL, and OPG. Based on this, it is considered a robust exposure factor compared to TNFa and LPS alone [23,24,56].

Regarding the GO enrichment analysis and the biological processes involved, the statistically overexpressed proteins were found to be associated with inflammatory and immune responses, as well as with the aging process. Specifically, the increased expression of certain proteins, such as the pro-inflammatory IL-6, which was observed in the present investigation, have been associated with the aging process and appear to be related to the frailty of the elderly, geriatric syndromes, and chronic systemic diseases [57]. Also, the increased serum level of the anti-inflammatory IL-10 has been detected in older people and is associated with the increase in IL-6 and the effort of the human immune system to regulate the expression of the latter [58]. Remarkable is that the expression of these proteins must be evaluated cooperatively to correlate their dysfunction with aging [59].

Regarding the monocyte chemoattractant proteins MCP-1 and MCP-2, these are members of the C-C chemokine family and a potent chemotactic factor for monocytes. MCP-2, which shares more than 60% sequence homology with MCP-1 and MCP-3 and about 30% homology with macrophage inflammatory protein (MIP)-1alpha, regulates the activation of normal T cell-expressed (RANTES), and MIP-1beta [60]. The upregulation of these proteins has been linked to the frailty of older people and morbidities associated with old age. Meanwhile, as reported by Matthew J. Yousefzadeh et al. in a paper on genetic and pharmacologic interventions, anti-aging treatments were aimed at reducing MCP-1 serum levels, a fact that proves the important role of this protein in the aging process [61]. The clinical impact, regarding these indications, is that monocyte chemoattractant proteins can be used as indicators of the biological age of the human organism and their regulation can be a target of therapeutic mediation for diseases related to aging, like Alzheimer’s disease, rheumatoid arthritis, and diabetes [62,63,64,65].

According to gene co-expression analysis that shows the level of confidence that two proteins are functionally associated based on the RNA-expressing levels in humans, the higher co-expression score was found in the present study for the pairs CCL3-CCL4 (score: 0.974) in the GCF group and CXCL9–CXCL10 (score: 0.848) in both the GCF and TNFa groups. C-C motif chemokine 3 (CCL3) and C-C motif chemokine 4 (CCL4) are monokines with inflammatory and chemokinetic properties belonging to the intercrine beta (chemokine CC) family. Both CCL3 and CCL4 bind CCR5 (CC motif chemokine receptor 5). C-X-C motif chemokine 9 (CXCL9) is a cytokine that affects the growth, movement, or activation state of cells that participate in immune and inflammatory responses. C-X-C motif chemokine 10 (CXCL10) is chemotactic for monocytes and T-lymphocytes. Both belong to the intercrine alpha (chemokine CxC) family and bind to CXCR3. The above-mentioned chemotactic cytokines form a diverse group of polypeptides with various functions [66]. These functions encompass drawing immune cells to infection and inflammation sites and promoting cell growth. Consequently, they act as agents against microbes and are crucial in the body’s defense against pathogens. Nevertheless, their capacity to attract white blood cells and enhance or extend the inflammatory reaction could significantly impact the development of oral conditions like chronic periodontitis, which often leads to widespread tissue damage. Cytokines play a crucial role in nearly every phase of periodontal disease development [67]. These substances can even be found in non-diseased areas. Beyond their function in guiding immune cells to infection sites, they also govern processes like the formation of new blood vessels, cell growth, programmed cell death, and the migration of tumor cells. Furthermore, it is becoming more evident that persistent inflammation plays a pivotal role in the advancement of oral tumors. Overall, cytokines are directly related to chronic inflammation in the oral cavity and the enrichment of these factors in this investigation confirms the acquirement of an SASP profile [68,69,70].

In conclusion, the experimental setup employed in the present study aimed to recapitulate young and aged oral tissues and to assess the impact of a panel of pro-inflammatory signals commonly present in the oral environment on the induction of cellular senescence and the initiation of the inflammation process. The two most common dental diseases, periodontitis and caries, promote an inflammatory environment in the oral cavity, whose initiation already from a young age and for a long period can lead to the secretion of more pro-inflammatory factors from the ever-growing senescent cells. In this condition, the repair and defense mechanisms of the human body decrease, while there is an increased risk for the spread of inflammation. The results show that indeed both at the genetic and at the proteomic level, a chronic inflammation can accelerate the cellular senescence process, especially at long-term exposure conditions.

This implies that inflammageing in the oral cavity can be associated with diseases that may spread systematically, so actions aimed at eliminating chronic inflammation in the oral cavity from an early age are deemed necessary. In the future, biomarkers that take part in the process of inflammageing and that emerged through this research (e.g., SASP profile) could serve as indicators to assess the existence of oral pathogenesis due to inflammation and lead to preventive measures against premature cell senescence that might accelerate ageing.

## 4. Materials and Methods

Establishment of Human Gingival Fibroblasts (HGFs) cultures

Human Gingival Fibroblast (HGF) primary cell cultures were established from gingival biopsies of three young healthy donors (20–24 years old) obtained during routine third molar extraction. This study was approved by the ethical committee of the Institutional Review Board (11/01.07.2020).

HGF cultures were established using enzymatic dissociation, as previously published [71]. Briefly, the cells were initially expanded in 25 cm^2^ flask (p. 0) in Dulbecco’s modified Eagle’s medium (DMEM, Invitrogen, Carlsbad, CA, USA) (Life Technologies, Thermo Fisher Scientific, Paisley, UK), supplemented with 10% fetal bovine serum- FBS (Life Technologies), 100 units/mL of penicillin, 100 mg/mL of streptomycin, and 0.25 mg/mL of amphotericin-B (all from Life Technologies) (=expansion medium) (CCM), and incubated at 37 °C in 5% CO_2_. Medium change was performed every 2 days. After reaching 70–80% confluency, cells were detached with a solution of 0.25% trypsin and 1 mM EDTA for 4–5 min and further subcultured in 75 cm^2^ flasks (p. 1).

Cell cultures were divided into two different experimental groups (G) according to the cell passage and the exposure period (short-term or long-term) to specific pro-inflammatory signals. The latter included exposure to one of the following: 1. human recombinant tumor necrosis factor alpha, TNFa (expressed in *E. coli*, Sigma-Aldrich, Darmstadt, Germany), 2. lipopolysaccharide, LPS (expressed in *E. coli*, Sigma-Aldrich), and 3. gingival crevice fluid, GCF, collected from deep inflammatory periodontal pockets of three systemically healthy adults. The GCF was characterized in terms of pro-inflammatory protein content by a DuoSet^®^ Human ELISA assay (Biotechne^®^, Minneapolis, UK), as described previously [23]. It was found to contain a cocktail of the following factors: IL-10: 242 (sd:14.7), BMP-7:66 (sd:9.2), TNFa: 145 (sd:14), RANKL: 146 (sd:11.8), OPG: 32.23 (sd:3.9), RANKL/OPG Ratio: 4.3 (sd:0.6), measured in pg/30 s.

The two experimental setups were as follows:

Group (G) 1. Late-passage cells exposed short-term to pro-inflammatory stimuli

HGFs expanded up to late passage (p. 10) were split into five experimental groups (G) according to treatment received, as follows:

- G1a received no treatment (negative control group).
- G1b was treated with two concentrations of TNFa (TNFa-1 = 0.1 μL/mL and TNFa-2 = 1 μL/mL).
- G1c was treated with two concentrations of LPS (LPS-1 = 0.2 μL/mL and LPS-2 = 1 μL/mL).
- G1d was treated with two concentrations/dilutions of GCF (GCF-1 = 5 μL/mL and GCF-2 = 10 μL/mL).
- G1e was treated with two concentrations of ethanol EtOH (positive control group) (EtOH-1 = 100 mM and EtOH-2 = 500 mM).

Specifically, HGFs were cultured up to p. 10 and then detached (0.25% trypsin/1 mM EDTA, 4–5 min) and replated in twelve-well plates at a density of 2.5 × 10^4^ cells/cm^2^. Cells in triplicates were exposed to each of the above-mentioned pro-inflammatory signals for a single exposure for 3 days, while cells not exposed to any signal served as the negative control and cells exposed to ethanol (EtOH) as the positive control. The experiment was repeated three times.

G2 early-passage cells exposed long-term to pro-inflammatory stimuli

This experimental setup included HGFs at passage 2 that were subjected to long-term exposure to the following pro-inflammatory factors:

- G2a received no treatment (negative control group C).
- G2b was treated long-term with 1 μL/mL TNFa.
- G2c was treated long-term with 0.2 μL/mL LPS.
- G2d was treated long-term with 10 μL/mL GCF.

Specifically, HGFs cultured up to p. 2 were detached (0.25% trypsin/1 mM EDTA, 4–5 min) and replated at a density of 5000 cells/cm^2^. The cells were divided into the three experimental groups and the control. Cultures of each group were passaged every four days. After 24 h of a new passage, the cells were exposed to each of the above-mentioned pro-inflammatory signals for 72 h, until the next passage. No medium change was performed until the next passage, so that the total exposure time of the cells to the above-mentioned stimuli would be 72 h. Cell passaging and/or treatment of the cells continued up to passage 10 (i.e., long-term exposure). Cells were counted at the beginning and the end of each passage with a hemocytometer (Neubauer cell chamber, Laboroptik, Lancing, UK) and plated at 5000 cells/cm^2^, in 100 mm diameter plates (Corning) for each of the passages/exposures. HGFs at early (p. 3–4), middle (p. 6–7), and late (p. 9–10) passages of this experiment were collected during this procedure to be further analyzed by the experimental assays described below. The experiment was repeated three times.

The biological assays performed for each of the experimental setups are described below.

Evaluation of cellular senescence

Cell lysates from all the experimental conditions were collected for real-time PCR analysis of senescence-related genes. Moreover, cells of each condition were processed for beta-galactosidase histochemical staining to evaluate cellular senescence.

Beta-galactosidase assay for evaluation of cellular senescence

Expression of senescence-associated beta-galactosidase activity (SA-beta-gal) was determined by a chromogenic assay kit (Sigma-Aldrich, Darmstadt, Germany), according to the manufacturer’s instructions. Briefly, cells were fixed in 4% paraformaldehyde (PFA), washed with PBS, and incubated with beta-Gal staining solution (40 mM citric acid sodium phosphate buffer, 1 M NaCl, 5 mM ferrocyanide, 5 mM ferricyanide, 2% DMF, 20 mM MgCl_2_, X-GAL 1 mg/mL in DMSO) for 14–16 h at 37 °C. Stained and unstained cells were counted under a light microscope (Zeiss, Axio Observer A.1) in five randomly selected optical fields of vision (×100) and the percentage of positive cells was calculated. Blinded subjective scoring of the percentage of blue-stained cells was used to quantify senescent cell fractions.

Assessment of senescence-related gene expression by real-time PCR arrays

Total mRNA was isolated from hGFs cultures exposed to pro-inflammatory pre-conditioning using the Nucleospin RNA isolation kit (Macherey Nagel, Germany) and reverse transcribed (1 μg/sample) using superscript first-strand synthesis kit (Takara, Japan) according to the manufacturer’s instructions. All reactions were performed using SYBR-Select PCR Master Mix (Applied Biosystems, CA, USA) in Step One Plus thermal cycler (Applied Biosystems), as previously described by our group [72]. Primers were designed using an online primer design tool (https://www.ncbi.nlm.nih.gov) (accessed on 18 November 2023) (Table 1). The senescence-related genes that were used to evaluate gene expression were *CCND1*, *SUSD6*, and *STAG1*. A standard melting curve was used to check the quality of amplification and specificity. The results were adjusted by amplification efficiency (LinRegPCR) and normalized against *GAPDH* as the housekeeping gene.

Analysis of SASP-related marker expression using an antibody proteome array

To identify SASP-related marker expression, cell lysates collected under the above-described pre-conditioning strategies were processed for SASP-related marker expression analysis using commercial and/or customized chemiluminescence-based antibody arrays (Human Inflammation Antibody Array Membrane 40 Targets, ab134003). These were performed according to the manufacturer’s instructions. Light signals were detected using a Chemiluminescence detection device (MicroChemi, DNR Bioimaging systems, Jerusalem, Israel). The detected spots were quantified using the “Dot Blot Analyzer” in the Image J software [73].

Bioinformatics analysis

The statistical significance levels of Tukey’s multiple post hoc tests were summarized in a matrix for all proteins and for all pairwise comparisons against the control group. A heatmap of the matrix containing the FDR values was built in R (R version 4.2.2). Protein names were mapped to UniProt IDs [74], followed by the automatic retrieval of comprehensive annotations that included the protein functions, interactions with other proteins, subcellular localization, gene ontology (GO) terms, and cross-reference identifiers to external pathway resources. STRING-DB [75] was used to build the interaction networks of the statistically significant proteins and to analyze co-expression patterns of the genes corresponding to the proteins of statistical interest. The relevance of each protein group with biological processes, molecular functions, and cellular components was assessed based on the assigned GO terms, following a pooling step to cope with multiple assignments for each protein. Cleveland plots were built on the most frequent GO terms per protein group.

Statistics

All experiments were run in two to four replicates and repeated at least three times. Statistical analysis of the data employed one-way analysis of variance (ANOVA), while multiple comparisons between groups for each of the biological endpoints under investigation were performed with Tukey’s post hoc test, using Prism 8.0 software (GraphPad, CA, USA). (* *p* < 0.05 and ** *p* < 0.01). Data are expressed as mean ± standard deviation.

## Figures and Tables

**Table 1:** Primers designed for the real-time PCR analyses of several senescence-related genes and the respective amplicon sizes of the PCR products.

Gene Symbol	Forward (5′-3′)	Reverse (5′-3′)	Amplicon Size (bp)
*CCND1*	AGCTGTGCATCTACACCGAC	GAAATCGTGCGGGGTCATTG	113
*SUSD6*	TTAGCTGCCGTCTCAACGAG	CTGGTCACGCCTGCTATGAT	170
*STAG1*	GATTGCAGCTCCGTTGAAGG	GCCGACCATCGACCTAGTTT	125
*GAPDH*	GACAGTCAGCCGCATCTTCT	GCGCCCAATACGACCAAATC	104

## Data Availability

Data are available from the corresponding author upon reasonable request.

## Supplementary Materials

The following supporting information can be downloaded at: https://www.mdpi.com/article/10.3390/ijms25010005/s1.
