# Supplementary material for: Biological Effects of “Inflammageing” on Human Oral Cells: Insights into a Potential Confounder of Age-Related Diseases
Source: Int J Mol Sci. 2023 Dec 19;25(1):5. doi: 10.3390/ijms25010005 (PMC10778866; doi:10.3390/ijms25010005)
Supplement: Supplementary file 1 [file ijms-25-00005-s001.zip › ijms-2754350-supplementary.pdf]

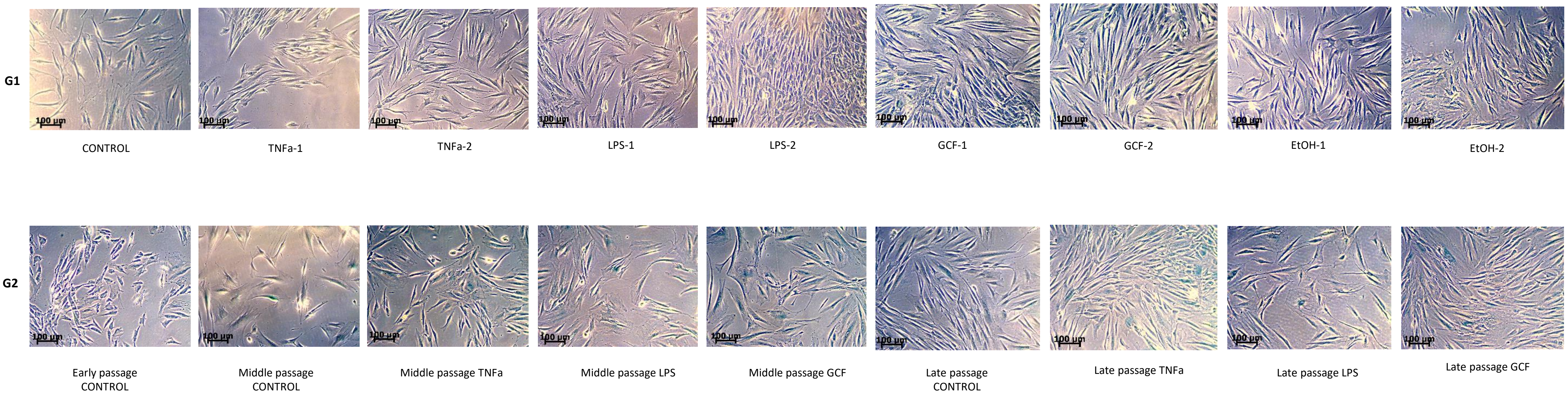

Supplementary Figure S1: Impact of long-term and short-term exposure of HGFs to several pro-inflammatory signals (TNFa, LPS, GCF) on β-galactosidase activity. Optical microscopy photographs of HGFs, exposed short-term and long-term to pro-inflammatory signals (sale bars: 100 µm).

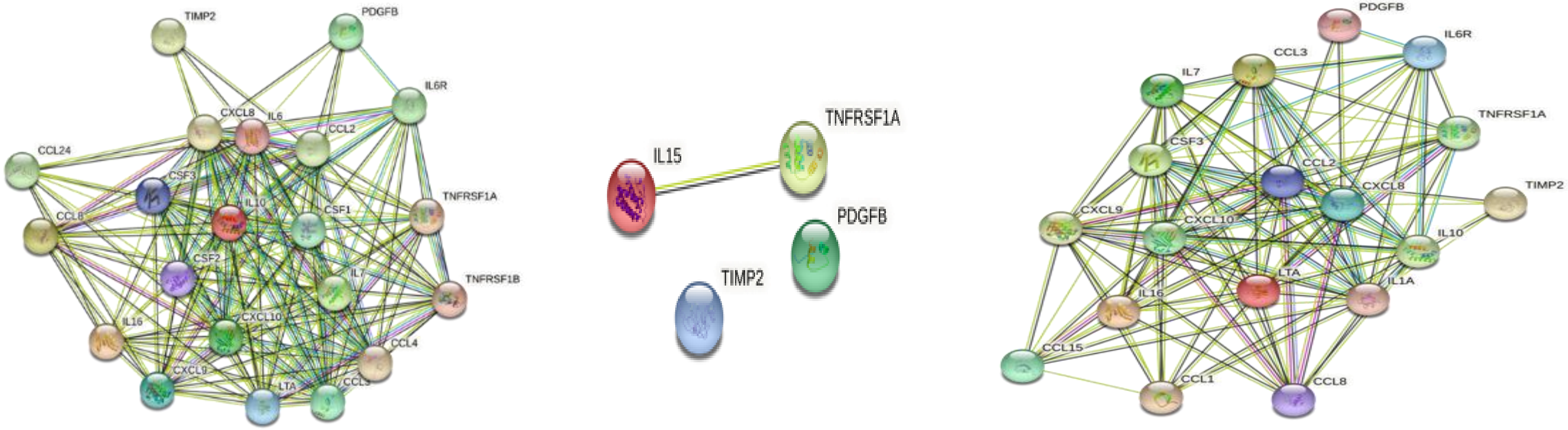

(a) (b) (c)

Supplementary Figure S2 : Interaction network of the statistically significant proteins (adj. *p*-value <= 0.05) for a) GCF, b) LPS, and c) TNFa. The line color indicates the type of interaction evidence.
